# The impact of source credibility and information quality on healthcare consumers’ attitudes: the moderating role of perceived severity

**DOI:** 10.3389/fpubh.2025.1689496

**Published:** 2025-12-04

**Authors:** Fan Chen, Ying Wang

**Affiliations:** 1 School of Economics and Management, Hubei University of Education, Wuhan, China; 2 School of Management, Wuhan Institute of Technology, Wuhan, China

**Keywords:** source credibility, information quality, healthcare consumers’ attitudes, perceived severity, healthcare service models

## Abstract

In the context of market competition, the acceptance of a doctor’s opinion by healthcare consumers depends on the source credibility and the information quality. In particular, differences in individual healthcare consumers’ perceptions of their illnesses may affect the choice of trusting the source credibility or the content of the information, i.e., even for minor ailments, they may prefer to trust a doctor with authority, regardless of the quality of the content of the information. By enhancing the perceived authority of doctors and the trustworthiness of information quality, communication effectiveness can be targeted to reduce consumer anxiety and eliminate communication barriers or upsets within a limited consultation time. In order to explore the underlying mechanisms of the association between source credibility and information content and consumers’ subjective attitudes, this paper takes healthcare services as an example, uses social support theory as the background, and based on the exhaustive likelihood model, firstly, applies structural equation modelling to examine the mechanism of the interaction’s influence on consumers’ attitudes, and then applies the multi-cluster structural model in conjunction with the protective motivation theory to examine the moderating effect of the perceived seriousness of the interaction on the influencing mechanism. The study found that interaction positively affects service satisfaction indirectly through the mediation of information quality and source credibility, and that perceived severity moderates the relationship between information quality and source credibility and consumer attitudes. In addition, the findings of the study provide theoretical guidance for the future exploration of the influence mechanism of consumer attitudes, and provide important insights into the innovation of online healthcare service models in the digital era.

## 1 Introduction

Nowadays, most people are dissatisfied with the medical service, because of a common phenomenon of “three long and one short,” such as long registration time, long waiting time, long time to take medicine and short time to see a doctor. The doctor often see a patient for only 3–5 min on average. The doctor’s judgment for the patient is simple, and the treatment is not explained in detail. With the development and popularization of Internet technology, people have increasingly easy access to a large amount of information and social communication opportunities. However, there are also some drawbacks and limitations, such as information overload and the push of a large amount of low-quality information, which makes it difficult for users to effectively identify valuable information and renders them vulnerable to misleading (50). Persuasion usually occurs in the process of interpersonal communication, and persuasion in natural contexts is not a traditional one-way information flow but a two-way information flow process (52). In the medical consultation process where information asymmetry is relatively prominent, doctors usually deliver persuasive information to patients, and doctors also receive feedback from patients. Patients are easily influenced by Internet information, questioning the doctor’s diagnosis process, and thus failing to make correct decisions. Doctor-patient communication is an effective way to establish doctor-patient trust and improve medical quality (7).

People seek medical treatment to cure their physical or psychological discomfort, according to their perception of the disease severity. The mean severity of disease in patients who received medical treatment was much higher than in patients who did not receive medical treatment, and the severity was the most direct and important factor in whether patients received medical treatment (1, 2). Predicting intention to receive a seasonal influenza vaccination using Protection Motivation Theory (1). Due to differences in patients’ personality characteristics and understanding of relevant medical information, patients’ perception of the severity of the disease is usually different, and patients’ decision-making will be correspondingly different. Protection motivation theory (PMT) suggests that people protect themselves based on two appraisal factors – threat (severity, vulnerability, and income) and coping (self-efficacy, operational effectiveness, and cost) (3). When people perceive a higher degree of threat in severity and vulnerability, they have stronger motivation and willingness to act to protect (4, 5). Therefore, even for the same type of disease in the same service environment, patients have different degrees of severity of the disease.

In medical treatment, doctors and patients make diagnoses, and treatment decisions mainly rely on the communication between patients and doctors. Through communication, doctors and patients exchange information and formulate treatment plans. In addition, doctors persuade patients to accept the diagnosis and treatment plans. The elaboration likelihood model (ELM) is a dual process to change their attitudes through two approaches—central and peripheral (6). When consumers adopt the central route, they engage in elaborate cognitive processing of information; in contrast, when they take the peripheral route, they tend to rely more on heuristic cues or feelings for information processing (10). In this study, the Elaboration Likelihood Model (ELM) is introduced as a reference theory to facilitate doctor-patient communication. Specifically, information quality, which represents the details of diagnosis, is corresponding to the central route of consumers’ information processing, while source credibility, represented by the characteristics of doctors providing treatment, is corresponding to the peripheral route of consumers’ information processing.

Today, most people are dissatisfied with healthcare services due to the widespread phenomenon of “three long waits and one short consultation.” This refers to lengthy registration times, extended waiting periods in the clinic, prolonged medication pickup, and brief consultation durations. On average, doctors spend only 3–5 min per patient during each appointment. Diagnoses are often simplistic, and treatment plans lack detailed explanations. With the advancement and widespread adoption of internet technology, people now have easier access to vast amounts of information and greater opportunities for social interaction. However, certain drawbacks and limitations exist, such as information overload and the proliferation of low-quality content. This makes it difficult for users to effectively identify valuable information and increases the risk of misinformation (50). Persuasion typically occurs during interpersonal communication. In natural settings, persuasion is not a traditional one-way flow of information but rather a two-way process (52). During medical consultations, where information asymmetry is particularly pronounced, physicians typically convey persuasive information to patients while also receiving feedback from them. Patients, easily influenced by online information, may question the diagnostic process, hindering their ability to make sound decisions. Effective doctor-patient communication serves as a vital pathway for building trust and enhancing healthcare quality (7). Concurrently, social support theory posits that social support fulfills individual needs and assists problem-solving, thereby influencing interpersonal relationships (8). Within the interactive process of doctor-patient communication, positive interactions facilitate the comprehensive sharing of medical consultation information, treatment details, and health care insights between physicians and patients (9).

Individuals seek medical treatment based on their perceived disease severity. Patients who receive medical care exhibit significantly higher average disease severity than those who do not, making severity the most direct and critical factor influencing treatment decisions (1, 2). Utilizing Protective Motivation Theory to predict seasonal influenza vaccination intentions (1). Due to differences in patients’ personality traits and understanding of relevant medical information, their perceptions of disease severity often vary, leading to corresponding differences in decision-making. Protective Motivation Theory (PMT) suggests that individuals protect themselves based on two evaluative factors—threats (severity, susceptibility, and benefits) and coping (self-efficacy, operational effectiveness, and costs) (3). When individuals perceive higher threat levels regarding disease severity and susceptibility, their motivation and willingness to take protective actions increase (4, 5). Consequently, even within identical healthcare settings, patients with the same disease may hold differing perceptions of its severity.

During the medical process, doctors and patients jointly make diagnostic and treatment decisions, which primarily rely on communication between them. Through this exchange, information is shared and treatment plans are formulated. Additionally, physicians persuade patients to accept diagnostic and therapeutic approaches. The Elaborated Possibility Model (ELM) posits a dual-process mechanism for attitude change via two pathways: the central route and the peripheral route (6). When consumers engage the central route, they undertake thorough cognitive information processing; conversely, when adopting the peripheral route, they tend to rely more heavily on heuristic cues or intuition for information processing (10). In this study, the Elaborative Possibility Model (ELM) and Social Support Theory are introduced as theoretical frameworks. Using the interaction within the physician-patient communication process as the independent variable, source credibility and information quality as mediating variables, and attitudes toward seeking medical care as the dependent variable, we explore the mechanism through which physician-patient interaction influences patients’ attitudes toward seeking medical care. Specifically, information quality—the level of detail in the diagnosis—corresponds to the central route of consumer information processing, while source credibility—the characteristics of the treating physician—corresponds to the peripheral route.

In addition, the study focused on the persuasion mechanisms of doctor–patient interactions, using the multiple group analysis method to analyze illness severity perception impacting the process adjustment effect.

## 2 Theory and hypothesis

The Elaborative Possibility Model is a dual-processing framework that describes the process of information refinement: When information is highly detailed, individuals engage in central processing, carefully considering and scrutinizing relevant information. Attitudes formed through this deliberate central processing tend to be more enduring. When information is less detailed, individuals engage in peripheral processing, focusing more on convenient cues such as celebrity endorsements for products or services, or the charm and appeal of the information sender (11). Within the elaboration likelihood model, persuasion can influence attitudes simultaneously or independently through central and peripheral processing. Information delivered through both pathways presents individuals with new possibilities, prompting them to reassess and adjust their existing beliefs and attitudes. Drawing from scholarly research (12), this study employs information quality as the central route variable—encompassing the benefits of the treatment plan, associated costs and expenses, and the patient’s assessment of communication with the physician. Source credibility serves as the peripheral route variable, reflecting the patient’s evaluation of the physician’s professional competence, reliability, and other attributes. Social support refers to beneficial interpersonal communication that shields individuals from the adverse effects of stressful events. As a cognitive assessment of personal closeness and relationship quality, it significantly influences how people adapt to various social environments (13). Social support theory (SST) posits that social support fulfills individual needs and aids problem-solving, thereby shaping interpersonal dynamics. Social support denotes beneficial interpersonal communication that shields individuals from the adverse effects of stressful events. As a cognitive evaluation of personal closeness and relationship quality, it significantly influences adaptation to diverse interpersonal settings (13). Social support can be categorized into emotional support and informational support. Emotional support involves providing care, understanding, and empathy, while informational support entails offering useful information such as recommendations, advice, or knowledge (14). Social support may originate from partners, family members, friends, colleagues, neighbors, or the community (49). Different helpers provide operational social support through various types of resources to assist recipients in coping more effectively with their environment (51). During doctor-patient interactions, patients perceive social support from physicians, encompassing both emotional support and informational support conveyed through communication.

### 2.1 The relationship between interaction and information quality and source trustworthiness

Dagger et al. (15) proposed that interaction includes service personnel’s attitude, information exchange process, and intimacy of the relationship between service personnel and customers. Doctor–patient interaction is the transmission process of medical services from doctors to patients and information from patients to doctors. It is the interaction between people, including two-way communication and feedback, and the exchange of roles between doctors and patients. Trust and interdependence emerge through interactions between the two parties (16). Doctor–patient interaction promotes a good relationship between doctors and patients. A positive doctor-patient interaction can also drive the process of value co-creation (17). The more information exchanged between doctors and patients, the more information patients can transmit to doctors, strengthening doctors’ understanding of patients’ conditions. Based on the patient’s condition, doctors can provide more effective diagnosis and treatment plans, disease-related professional knowledge, and preventive measures advice. Simultaneously, patients can receive a more effective diagnosis and treatment information while experiencing information support from the doctor.

Many studies have shown that source credibility has always been an important factor in communication (18). However, due to medical services’ serious information asymmetry compared with other service industries, medical services have higher perceived risks. Thus, strengthening the interaction between doctors and patients, providing patients with emotional support such as care and understanding, improving mutual impression will promote a harmonious and intimate relationship between doctors and patients and enhance patients’ trust in doctors. Therefore, we propose the following hypotheses:

*H_1_*: Interaction has a significant positive effect on information quality.

*H_2_*: Interaction has a significant positive effect on source credibility.

### 2.2 The relationship between information quality, source credibility, and attitude

The formation of attitudes is not only based on rational cognitive judgment results but also includes behavioral experience and emotional reactions (19–22). In this regard, the elaboration likelihood model helps understand the formation of individual attitudes. The acquisition and processing of information are usually the basis of formation and change of attitudes, that is, to persuade individuals to change their attitudes through information processing and judgment of information sources.

In service communication, individuals’ judgment of information is a process of rational cognition. Positive information contains more credible content, more emphasis on beneficial results, and is more persuasive, while negative information is the opposite (23). The better the quality of the information provided by doctors, the more adequate and effective the information for patients’ conditions, and the more reasonable and powerful facts the information contains, the more persuasive it is to patients. However, in the case of poor information quality, the information content contains insufficient reasonable and beneficial information and lacks persuasion for patients.

The formation and change of attitude may come from the central approach or the peripheral approach. In communication, the information recipient will be more willing to intervene when the source of information is more credible (24). Source trustworthiness means that the recipient considers the source professional, objective, or reliable. Thus, the stronger the doctor’s professional ability in the communication process, the more positive the patient’s emotional response. Also, the stronger the patient’s trust in the doctor, the more positive the patient’s medical attitude. Customers’ trust in service providers can reduce uncertainty in services, reduce perceived risks, and influence decision-making, a pre-influencing factor of behavior. Based on the above literature analysis, we propose the following hypotheses:

*H_3_*: Information quality has a significant positive impact on medical attitude.

*H_4_*: Source Credibility has a significant positive impact on medical attitude.

### 2.3 Mediating effect of information quality and source credibility

Information quality and source credibility are important variables applied to the central and peripheral approaches of persuasion, respectively (6). Persuasion is the main way to achieve mutual benefits through interaction between service personnel and individuals. Persuasion motivates individuals to enhance their trust of doctors who provide diagnosis and treatment information, accept the doctors’ diagnosis and treatment plans, meet the desire to restore their health status, and improve patients’ attitudes towards medical treatment. The interaction between doctors and patients aims to improve patients’ perception of doctors’ service attitude, promote the intimacy between doctors and patients, exchange information, and motivate patients to accept the doctors’ reasonable diagnosis and treatment plan. Therefore, by enhancing the effectiveness of persuasion, doctor–patient interaction can positively impact doctor–patient attitude towards medical treatment, and persuasion becomes a mediator variable between doctor–patient interaction and patient attitude towards medical treatment.

When a doctor and patient establish a good interaction, the patient perceives social support from the doctor. Through doctor–patient communication, the patient gains information support, and the doctor can provide the patient with relevant medical knowledge, diagnosis, treatment suggestions, and matters needing attention after treatment. That is, the patient provides the detailed condition for the doctor, and the doctor provides the perfect diagnosis and treatment plan information for the patient. The more effective the arguments in the communication, the more effective the persuasion effect influencing the patient’s attitude towards medical treatment.

Through good interaction between doctors and patients, each gains the necessary information and emotional support, and doctors provide care and understanding for patients. Through emotional support, doctors and patients can maintain a good relationship where patients can get emotional recognition, emotional resonance, and reduce their tension and anxiety caused by the uncertainty of medical service, thus enhancing their trust in medical service personnel. Studies have shown that trust can reduce perceived risk (25), strengthen source credibility in medical services, and improve uncertainty through trust, thus affecting patients’ attitudes towards medical treatment. Furthermore, trust can trigger positive emotions, conducive to maintaining mutual communication between doctors and patients and promoting positive behavioral intentions and attitudes. Based on the above literature analysis, we propose the following hypotheses:

*H_5_*: Information quality plays a mediating role between interaction and medical attitude.

*H_6_*: Source credibility has a mediating effect between interaction and medical treatment attitude.

### 2.4 Moderating effects of perceived severity on the relationship between information quality, source credibility, and medical treatment attitude

When faced with some health threat, such as a new symptom or diagnosis, people will build a subjective perception of the health threat based on relevant medical knowledge and previous relevant experience (26). People base their subjective perception of illness on implicit information or relevant common sense, which can affect the mental health of patients and their ability to cope with illness (27). Perceived severity and perceived probability have always been two important attributes of health hazards, and they interact to influence people’s health protection behaviors (28).

Many scholars have carried out relevant studies on how patients’ perceived severity of different diseases affects their medical treatment behavior. For example, Meikle (29) found a high correlation between patients’ perceived severity rating of tinnitus and the frequency of sleep disturbance. In addition, Helgeson (30) suggests that the stronger the perception of the severity of chronic disease threat, the stronger the relationship between disease control and adjustment. In another study, Petrie and Weinman (26) suggest that patients with stronger beliefs about their illness improve doctor–patient communication and outcomes. Patients’ perceived severity significantly predicts their mental health status. The more severe the perception, the more uncertain the treatment outcome, and the greater the pressure patients feel. This, in turn, affects individuals’ assessment of physical or psychological dangers, leading them to experience lower levels of security (31–33).

Compared with patients who treat their illness positively, patients with a negative view of their illness experience a more severe impact. The more serious the perception of their illness, the stronger the uncertainty of their illness.

The central approach is the information processing approach which involves more cognitive effort. When patients have a higher degree of disease severity, their subjective perception is stronger. Their emotions will include more nervousness, anxiousness, irritability, and other psychological states. Thus, patients might invest less in the rational cognitive process in medical treatment. In particular, there is serious information asymmetry in medical services. It is difficult for patients to judge the reliability and professionalism of information from doctors by relying on their personal knowledge of medical expertise. The judgment of information professionalism depends on the doctors’ expertise, such as the doctor’s professional title.

The peripheral approach involves less cognitive heuristics in information processing and is more likely to favor the trusted expert messenger. Compared with patients with weak perceived severity, patients with a more severe subjective perception of their illness have stronger uncertainty and risk perception that may affect the treatment process and results. When the perceived risk is high, people will avoid the risk to obtain greater benefits (34, 35). Social judgment theory suggests that individuals with higher involvement tend to show more negative evaluations of communication and rejection (36). When the information source is more trustworthy, patients’ trust in doctors is stronger, reducing patients’ negative emotions and risk perception. Thus, we propose the following research hypotheses:

*H_7_*: The higher the perceived severity of illness, the less the influence of information quality on medical treatment attitude.

*H_8_*: The higher the perceived severity of illness, the greater the influence of source credibility on medical treatment attitude.

### 2.5 Research conceptual models

**Figure 1:**
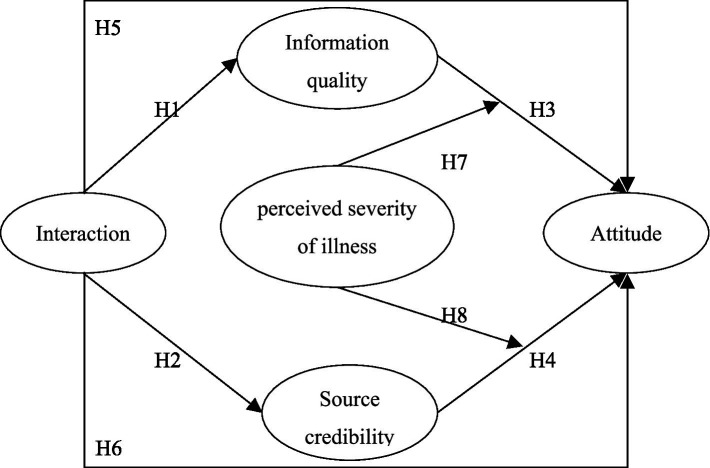
Elaboration likelihood model.

This study set up eight hypotheses to construct a conceptual model. The dotted line in Figure 1 shows the hypotheses of the moderating effect of perceived severity and the specific research hypotheses.

## 3 Method

### 3.1 Questionnaire

The five constructs of interest to this study were perceived Interaction, Source credibility, Information Quality, Attitude, Perceived Severity of Illness. All constructs were measured using multiple-item perceptual scales, using pre-validated instruments from prior research wherever possible, and reworded to relate specifically to the context of medical service. Interaction was defined as the communication feedback between patients and physicians during the diagnosis and treatment process. Interaction was assessed using Dagger et al.'s (15) nine-item Likert scale. Source credibility was assessed using Lee's (37) four-item Likert scale, where the physician serving as the information source during the medical visit was evaluated based on their competence and trustworthiness. Information quality was defined as the judgment regarding the exchange of information with the physician during the medical visit and was also assessed using Lee's (37) four-item Likert scale. Attitude was defined as the evaluation of the medical visit process. Attitude was measured using four Likert scaled items developed and validated by Davis (38). We measured each item using a seven-point Likert scale that measured respondents’ attitudes, ranging from 1 (completely disagree) to 7 (completely agree).

Past studies often measured the severity of the disease objectively using tools such as APACHE II as the primary criteria for patients’ enrollment in hospitals and doctors’ diagnosis and treatment decisions (39). Vincent et al. (40) proposed that the objective measurement of severity score scale would lead to deviation of score results due to age, time, and other factors, which is not suitable for medical decision-making. Therefore, we adopted a subjective measurement in our study, using a seven-point scale and referring to the disease severity measurement method in the CHNS database to measure the topic “What do you think of the severity of the disease?” The scale ranged from 1 (*a very small problem*) to 7 (*a very serious problem*). The subjective measurement scale instead of objective measurement is suitable for this study and convenient for empirical analysis.

### 3.2 Data collection

We mainly invited research companies that conduct research activities and collect data online. The Data were collected for 15 day online. The respondents are the groups who have had seen a doctor in the past year. We collected 404 valid questionnaires. The respondents are 218 males and 186 females.

The characteristics of the valid questionnaire sample are shown in Table 1, from the point of view of the gender of the users, there are more males than females, accounting for 54.0 percent of the total sample; the age is mainly concentrated in 25–44 years old, accounting for 76.2 per cent; the highest education level of the family members is mostly bachelor’s degree, with 223 people, accounting for 55.2 percent; and the place of residence is concentrated in the big cities, with 190 people, accounting for 47.0 percent. The characteristics of the research sample in terms of gender, age, education and place of residence are basically the same as those of previous studies, indicating that the sample better reflects the basic characteristics of the respondents.

**Table 1:** Descriptive analysis of demographic variables characteristics (unit: person).

Characteristic		Frequency	Percentage	Characteristic		Frequency	Percentage
Gender	Male	218	54.0%	Education	Primary School or Below	3	0.7%
					Secondary School or Technical Secondary School	39	9.7%
	Female	186	46.0%		Junior College	103	25.5%
					Undergraduate Degree	223	55.2%
					Postgraduate or Above	36	8.9%
Age	24 years old and below	24	5.9%	Residence	Big City	190	47.0%
	25–34 years old	156	38.6%		Medium City	118	29.2%
	35–44 years old	152	37.6%		Small City	79	19.6%
	45–54 years old	63	15.6%		Township and Surrounding Areas	15	3.7%
	55 years old and above	9	2.2%		Rural Area	2	0.5%

## 4 Data analysis

### 4.1 Scale reliability and validity

This study first used confirmatory factor analysis to test the dimension of the measurement scale (41). Second, we set the four variables of medical attitude, information quality, source credibility, and interaction to correlate freely and then conducted confirmatory factor analysis. To improve the reliability and validity of the scale, we deleted the items that affected the scale performance according to the results of confirmatory factor analysis and component reliability. Table 2 shows the final measurement items of each dimension, including factor analysis results: CMIN/DF = 1.866, NFI = 0.966, TLI = 0.980, CFI = 0.984, GFI = 0.948, RESMA = 0.046. A load of each factor was significant at the 0.001 level and greater than 0.5, indicating that the scale had good concentration validity.

**Table 2:** Confirmatory factor analysis results.

Scale item	Item loading	Mean	S.D.
Attitude		AVE = 0.691	CR = 0.870
A1 Good idea to seek treatment at this hospital	0.818***	4.94	1.162
A2 Wise choice to seek treatment at this hospital	0.815***	5.00	1.217
A3 Satisfied with the medical care I received at this hospital	0.860***	5.00	1.206
Information quality		AVE = 0.608	CR = 0.861
IQ1 The information provided by the doctor is comprehensive	0.759***	4.89	1.145
IQ2 The information provided by the doctor is helpful	0.763***	5.05	1.113
IQ3The information provided by the doctor is valuable	0.810***	5.10	1.141
IQ4The information provided by the doctor is persuasive	0.785***	5.01	1.117
Source credibility		AVE = 0.637	CR = 0.840
SC1The doctor is highly knowledgeable in this field	0.802***	5.00	1.208
SC2 The doctor is trustworthy	0.789***	4.99	1.218
SC3The doctor is an expert in this field	0.803***	4.94	1.213
Interaction		AVE = 0.684	CR = 0.912
I1 Hospital staff always listen to what I say	0.821***	4.87	1.280
I2I feel that the hospital staff can understand my needs	0.791***	4.90	1.205
I3 Hospital staff can explain things to me in a way I can understand	0.855***	4.89	1.182
I4 Hospital staff are willing to answer my questions	0.821***	4.98	1.238
I5I trust the hospital staff care about me	0.824***	4.82	1.290

***p < 0.01, **p < 0.05, *p < 0.1.

After verifying the dimension of the scale, we tested the reliability and validity of the scale to ensure the results’ authenticity and applicability. This study used component reliability to test the reliability of the scale. The component reliability of each dimension was over 0.8, indicating that the scale had good internal quality, high consistency, stability, and small measurement error (Table 2).

First of all, since the measurement items in study are from previous research literature, this guarantees content validity (42). Second, we calculated the reliability and average variation of latent variables extraction (43). The potential of variable composition of reliability (CR) in this study is more than 0.8, and the average variation (AVE) extraction quantity value is more than 0.6 (Table 1). Thus, the variable has good internal consistency, and the measurement model has good convergent validity.

To assess the discriminant validity, we run two CFAs on each pair of scales (44). In the first analysis of each pair of scales, the two constructs were allowed to freely correlate (i.e., unconstrained model). In the second, the correlation between the two constructs was set to 1 (i.e., constrained model). As shown in Table 2, the χ^2^ difference in each model, constrained and unconstrained, are significant (*p* < 0.01), which suggests that constructs demonstrate discriminant validity.

Table 3 shows the calculation results of the chi-square difference test. The differences among all chi-square values are significant, indicating that different dimensions have good discriminability and the scale has good discriminant validity. Thus the scale in this study has good one-dimensional reliability, good internal consistency and reliability, and good structural validity—the scale is suitable to study the relationship between variables in this study.

**Table 3:** Pairwise CFA tests of measurement scale discriminant validity.

Construct scale pairs		Unconstrained model		Constrained model		χ^2^ difference
		χ^2^	df	χ^2^	df	
Attitude	Information Quality	39.63	13	70.62	14	30.99
	Source credibility	21.48	8	29.50	9	8.02
	Interaction	27.70	19	103.59	20	75.89
Information quality	Source credibility	54.02	13	84.42	14	30.40
	Interaction	37.96	26	203.44	27	165.48
Source credibility	Interaction	34.78	19	110.97	20	76.19

All χ^2^ differences are statistically significant at *p* < 0.001 level.

### 4.2 Hypothesis testing

This study employs structural equation modeling (SEM) for hypothesis testing, primarily based on two considerations: (1) SEM can simultaneously handle both observed and latent variables, effectively controlling measurement error; (2) SEM is highly suitable for testing complex path models involving mediating effects (45). This aligns well with the research objective of this study, which aims to explore underlying mechanisms.

This research established the model, used the path analysis, and fit the index to show χ^2^/DF = 2.649, NFI = 0.951, TLI = 0.962, CFI = 0.969, GFI = 0.927, and RESMA = 0.064. The fitting results are good. These results support H_1_ to H_4_. Table 3 shows the path coefficient and path method. The results showed that the interaction between medical staff and patients had a significant positive impact on information quality and source credibility. Information quality and source credibility had a significant positive impact on attitude.

Path coefficients results show that information quality (*β* = 0.435, *p* < 0.001) and source credibility (*β* = 0.608, *p* < 0.001) both have significant positive impacts on attitude, thus H1 and H2 are supported; interaction has significant positive effects on information quality (*β* = 0.710, *p* < 0.001) and source credibility (*β* = 0.842, *p* < 0.001), and H3 and H4 are supported.

### 4.3 Mediation effect analysis

**Figure 2:**
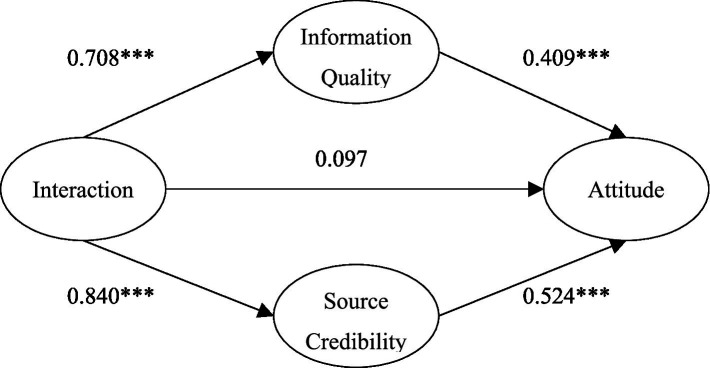
The nested model.

In the influence of interaction on attitude, information quality and source credibility may have mediating effects. Thus, we used the nested model to study the mediating effects. Based on the theoretical model, we added the direct influence path of interaction on attitude to form a nested model (see Figure 2) to verify whether there is a direct influence relationship between interaction and attitude.

The path analysis shows that the path coefficient of interaction on attitude is not significant in the nested model (Figure 2, *β* = 0.097, *p* = 0.368); the interaction had no direct effect on attitudes. At the same time, we compared the fitting index of the nested model and the theoretical model. We found no significant change between the nested model and the theoretical model (nested model fitting results: χ^2^/DF = 2.649, NFI = 0.951, TLI = 0.961, CFI = 0.969, GFI = 0.928, RESMA = 0.064, △χ^2^ = 0.965). This result indicates that interaction has no significant direct impact on medical treatment attitude. The results also showed that information quality and source credibility had an indirect effect on the relationship between interaction and medical treatment attitude—a completely mediating effect. The interaction between doctor and patient had a complete mediating effect on medical treatment attitude through information quality and source credibility (the mediating effect was 0.29 and 0.44, respectively). These results support H_5_ and H_6_.

In this study, robustness testing of the model was conducted using substitution tests and exclusion of outlier samples, among other methods. First, robustness testing was conducted using the Bootstrap method, employing the Medcurve macro developed by Hayes and Preacher with 5,000 resampled iterations and a 95% confidence interval. Credibility was found to exert a significant mediating effect in the interaction between influence and attitude. The regression results from the robustness test aligned with those from the path analysis. Second, considering the distinctive characteristics of highly educated groups in information perception, the “highly educated” cohort was excluded during re-estimation. Results indicated that all path coefficients remained significant with good model fit. Thirdly, accounting for the relatively constrained healthcare resources in rural areas, re-estimation was conducted excluding groups with poorer healthcare access such as “townships and communities” and “rural areas.” Results indicated that all path coefficients remained significant with good model fit. The aforementioned test results collectively demonstrate that the mediating mechanism of information quality and source credibility in the interactive influence of attitudes is highly robust, rendering the research findings reliable.

### 4.4 Multiple group analysis

This study used multi-group analysis to test the influence of different perceived disease severity on the model. First, we used a single-item scale to measure the severity ranging from 1 (*very small problem*) to 7 (*very serious problem*). Then, we summed and averaged the scores of each respondent. We regarded those greater than the mean as the group with high severity (*n* = 247) and those less than or equal to the mean as low severity (*n* = 157). This approach is in line with the psychological characteristics of the medical crowd; most of those who go to the hospital for medical treatment have a serious perception of their condition.

Multi-group structural equation model analysis tests help evaluate whether the same model is suitable for different populations in a sample. Thus, we can consider the variables used for grouping as moderating the hypothetical model (43). First, we set the measurement model; and limit the same parameters of the regression coefficients of the groups with high and low perceived severity of illness. We assumed that the default model was true, and we compared the default model with the constraint model, *p* = 0.002 < 0.01. The results indicating that the measurement model coefficient of the two groups with different severity has a significant difference at the significance level of 0.01, and perceived severity of illness has a moderating effect on the measurement model.

**Figure 3:**
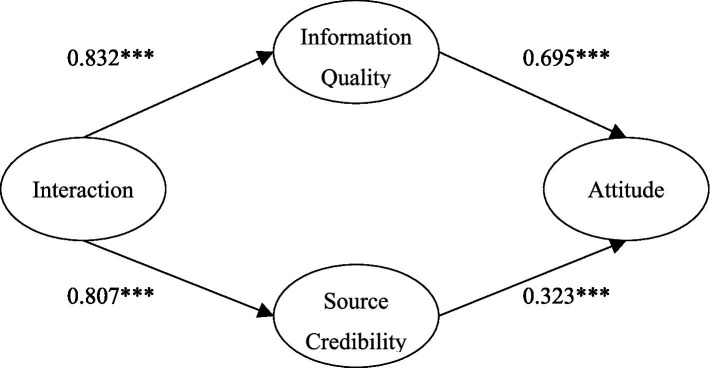
The model of the impact of interaction on medical attitude under low perceived severity.

**Figure 4:**
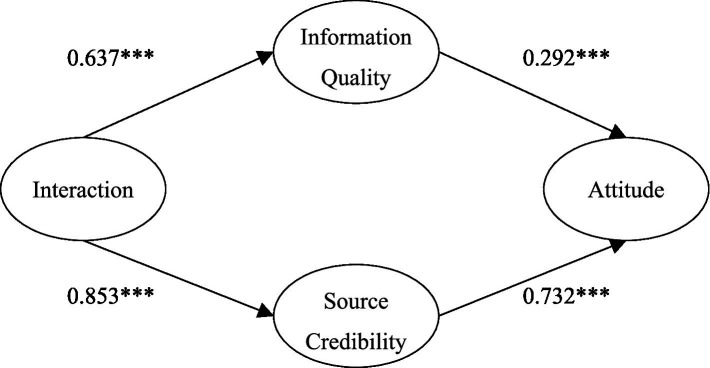
The model of the influence of interaction on medical attitude under high perceived severity.

The above illustrates the overall phenomenon and indifference test. Further, we used parameter pairing to investigate whether the specific factor load blinded the effect between the two groups. We observed the critical ratio value of the coefficient of the measurement model between the two groups. The results showed that the absolute values of the critical ratios of information quality and source credibility on attitude were 3.313 and 3.43 greater than 3.29, indicating that at a significance level of 0.001, the influences of information quality and source credibility on attitude were significantly different. The results showed that the absolute values of the critical ratios of interaction on information quality and source credibility on attitude were 2.276 and 0.529 lower than 3.29, indicating that at a significance level of 0.001, the influences of information quality and source credibility on attitude were non-significantly different. By observing the path coefficients of different perceived severity of illness, we found that the higher the severity of illness, the less the impact of information quality on attitude (*β* = 0.292 < *β* = 0.695), and the greater the impact of trusted sources on attitude (*β* = 0.732 > *β* = 0.323). In other words, compared with patients with low severity, the peripheral approach has a greater influence on attitude in patients with high severity. Therefore, the relationship between different perceived severity, information quality, source credibility, and attitude is significantly different. This finding supports H_7_ and H_8_ (Figures 3, 4).

We also investigated the moderating effect of perceived severity. The results showed that perceived severity positively moderated the relationship between central information quality and attitude and negatively moderated the relationship between external information source credibility and attitude. When the severity of patients’ perception is higher, the influence of source credibility on attitude is greater, and the influence of information quality on attitude is smaller. This finding indicates that patients pay more attention to the credibility of the information source in the communication process; that is, patients pay more attention to the characteristics of the doctors providing the diagnosis and treatment plan, professional ability, reliability, etc. Conversely, when the perceived severity of illness is low, the influence of trusted sources on attitude is smaller, and the influence of information quality is greater. Patients pay more attention to the benefits of the diagnosis and treatment plan, the cost and expense they need to pay for the doctors’ information, and their judgment of the information from doctors.

### 4.5 Heterogeneity test

#### 4.5.1 Differences in medical institutions for treatment and form of visits, Availability of supplementary health insurance

Tertiary hospitals have a standardized consultation process, centralized resources, and patient interactions focus on efficiency, with patient attitudes affected by both technological trust and lengthy processes; non-tertiary hospitals have flexible services, fast waiting times, and more individualized interactions, but patient attitudes are prone to fluctuations due to convenience at the primary level, technological concerns, or referral needs (Table 4).

**Table 4:** Heterogeneity test.

Path	Triple A	Non-Triple A	Hospitalization	Outpatient service	Attend	Not attend
Interaction → Source Credibility	0.877 (***)	0.959 (***)	1.005 (***)	0.899 (***)	0.964 (***)	0.872 (***)
Interaction → Information Quality	0.826 (***)	0.910 (***)	0.987 (***)	0.834 (***)	0.934 (***)	0.811 (***)
Information quality → attitude	0.407 (***)	0.337 (0.016)	3.170 (0.512)	0.360 (***)	0.378 (0.025)	0.408 (***)
Source Credibility → attitude	0.631 (***)	0.685 (***)	−2.103 (0.660)	0.661 (***)	0.633 (***)	0.640 (***)

In order to study the impact of the choice of different visiting medical institutions on the mechanism of interaction and attitude, this study divided the sample into tertiary hospitals and non-hospital samples for multicluster regression analysis, and the regression results showed a chi-square value of 1.986 and a *p*-value of <0.001, indicating that there is a significant difference between the two groups. The results showed that the quality of information was more able to significantly improve their attitudes in tertiary hospitals compared to non-tertiary hospitals.

Inpatient patients are more likely to be affected by emotional connection and systematic care experience due to long-term patient-physician contact and in-depth interdepartmental collaboration; outpatient patients are limited by high-frequency and fragmented consultation patterns, and patient-physician interaction focuses on immediate problem solving, so their attitudes towards medical care are more dependent on the efficiency of information transmission and immediate feedback of diagnosis and treatment results.

On the basis of the above results, this study analyses the heterogeneity of the mechanisms of different forms of consultation. In this study, the samples were divided into inpatient and outpatient samples for multi-cluster regression analysis, and the regression results showed that the chi-square value was 2.044, with a *p*-value of <0.001, indicating that there was a significant difference between the two groups. The results of the regression coefficients showed that the quality of information and credibility of the source of outpatient process were significantly able to improve attitudes compared to ambulatory.

According to the existing employee and resident medical care, patients with supplementary medical insurance pay more attention to the quality of service and the depth of doctor-patient communication due to the alleviation of economic pressure, and their attitude towards medical care is biased towards active participation and trust; whereas patients without supplementary medical insurance are sensitive to out-of-pocket costs, and their interactions are more focused on the efficiency of basic diagnosis and treatment and transparency of costs, and their attitudes are susceptible to the complexity of economic burden and reimbursement policies.

In this study, the sample was divided into those who participated in other health insurance and those who did not participate in other health insurance samples for multicluster regression analyses, and the regression results showed that the chi-square value was 1.949, with a *p*-value of <0.001, indicating that there was a significant difference between the two groups. The regression results indicate that the quality of information and credibility of the source for outpatient process information is more likely to significantly improve their attitudes compared to ambulatory.

## 5 Discussion

This study employs a detailed possibility model, utilizing information quality and source credibility as mediating variables, to investigate the influence pathways of doctor-patient interactions on patients’ healthcare choice behavior, alongside the moderating effect of perceived severity on these pathways. This contributes to enriching the literature within the persuasion and attitude change domain, refines the deep processing possibility model, and establishes a more comprehensive framework of influencing factors and pathways for persuasion and attitude change. It holds considerable reference value for subsequent theoretical research and targeted practical implementation. Findings indicate that both clinicians and patients process and interpret each other’s information in real-time while simultaneously anticipating and projecting reciprocal responses. Positive interactions between healthcare providers and patients significantly enhance information quality and source credibility, thereby amplifying persuasive effects through both central and peripheral processing mechanisms. Concurrently, patients exhibiting strong self-awareness and pronounced emotional responses require more credible source information, whereas those with weaker self-awareness and less evident emotional reactions place greater emphasis on information quality.

Based on these primary findings, this paper proposes three-pronged countermeasures: Firstly, optimize the content of doctor–patient interactions to enhance information quality and source credibility. Within healthcare services, strengthen effective communication between practitioners and patients. Encourage healthcare professionals to provide patients with more detailed diagnostic and therapeutic information, propose appropriate personalized diagnostic and treatment plans, and offer care and understanding while providing emotional validation and empathy. This reduces tension and anxiety arising from healthcare uncertainties and builds patient trust in healthcare providers. Through interaction with healthcare providers, patients can make informed treatment decisions and actions, moving beyond a passive role in the consultation process. This approach more effectively addresses their individualized and unique needs. Increasing the frequency of doctor-patient interactions can also enhance patients’ access to informational support within their social support networks (46). Research indicates that doctor-patient interactions not only afford physicians valuable opportunities but also cultivate patients’ Natural Quotient (NQ). Grounded in Granular Interaction Thinking Theory (GITT), NQ is defined as the capacity to perceive, process, and organize information about ecological interconnections—thereby fostering deeper ecological consciousness and guiding sustainable behavior (47). Within healthcare services, scientifically guiding patients to comprehend the symbiotic relationship between humanity and nature—particularly green spaces and blue spaces—can transform ecological cognition into health-promoting behaviors and sustainable lifestyles. This approach cultivates patients’ NQ, fostering connections between nature and health. It not only enhances patients’ physical and mental wellbeing but also establishes appropriate interaction with nature as a low-cost, highly accessible preventive health intervention. Nature itself thus becomes a low-cost preventive “medicine.” This approach not only reduces readmission rates and hospitalization due to emotional issues or poorly managed chronic conditions but also effectively alleviates the economic burden on healthcare systems and the psychological strain on medical staff.

During consultations, healthcare professionals should enhance the ecological health relevance of information by linking patients’ conditions to the health benefits of green and blue spaces, providing concrete and verifiable data. Through professional science communication, the health benefits of green and blue spaces are explained tangibly. Patients are systematically informed about the physiological advantages of green spaces—such as forests and community green areas—in lowering blood pressure and reducing stress hormones, alongside the unique value of blue spaces—like oceans and lakes—in improving sleep quality and enhancing emotional regulation. Simultaneously, personalized nature interaction plans are developed based on patients’ living contexts, activity capabilities, and natural preferences. For instance, these plans may incorporate chronic disease conditions to reinforce the cognitive link between nature and health. Secondly, to bolster the credibility of information sources, clinicians must proactively demonstrate authoritative references for relevant professional content, thereby enhancing patient trust. For patients who process information through peripheral channels, both positive reputation and emotional support can promote their value co-creation behavior (48). During consultations, cite relevant reports published by the World Health Organisation, share clinical case studies from authoritative medical institutions, and invite community ecologists or patients who have participated in nature-based health interventions to contribute to the dialogue. This dual-source approach—combining clinical expertise with third-party practical experience—reinforces the reliability of information, encouraging patients to accept and implement treatment recommendations.

Secondly, tailored interaction strategies prioritize emotional reassurance, tiered information delivery, and precise needs matching according to variations in patients’ self-perception intensity and emotional reactivity. This approach aligns with the study’s findings on regulatory effects while laying foundations for cultivating patients’ natural quotient (NQ). For patients exhibiting heightened self-perception and pronounced emotional responses, the primary focus is on reinforcing source credibility. This is achieved by demonstrating the professional authority of healthcare personnel through multiple dimensions, thereby alleviating intense emotions triggered by fears surrounding treatment uncertainties. Specifically, healthcare providers may proactively share evidence-based case studies of similar patients relevant to their condition, cite World Health Organisation data, present their professional qualifications, or reference locally developed natural health guidelines co-published by primary care institutions and community organizations. By integrating professional credentials, authoritative case studies, and localized data, this approach establishes credible information sources that reduce patient anxiety and builds trust in healthcare services. For primary care institutions with limited specialist resources, this shortfall can be addressed through remote consultations with domain experts, ensuring the credibility of information transmission. Secondly, for patients with weaker self-awareness and less pronounced emotional responses, the focus should be on enhancing the depth and quality of information provided. This involves delivering genuinely helpful and valuable insights, thoroughly explaining diagnostic and treatment processes. When explaining diagnostic outcomes and treatment plans, healthcare professionals should proactively link environmental factors to disease progression and incorporate the cumulative impact of natural elements when discussing health risks. Concurrently, such patients require verifiable information support—such as distributing authoritative manuals on nature-based recovery—to ensure scientific rigor and prevent poor treatment adherence stemming from insufficient disease awareness.

Thirdly, balancing healthcare resources, upgrading primary care capabilities, and integrating an ecological health orientation should guide patients towards proactively choosing primary care facilities. This approach alleviates pressure on major hospitals while establishing contextual platforms for cultivating patients’ natural quotient (NQ), thereby encouraging and directing them to seek care at primary healthcare institutions. ‘Medical care accounts for merely 8 % of one’s health; lifestyle, living conditions, and financial security play a greater role. We must adopt a more comprehensive perspective,’ stated Professor Qide Han at the 16th Annual Meeting of the China Association for Science and Technology. This underscores that primary healthcare institutions can adequately meet the needs of the majority of patients. The health people pursue encompasses not merely the absence of physical illness, but comprehensive wellbeing encompassing psychological and social adaptation. Health depends not solely on medical care, but more significantly on lifestyle, public health, social and natural environments, economic conditions, and genetic factors. For instance, experts entering communities to disseminate information on healthy lifestyles and living conditions, helping patients increase their health knowledge and alter their health behaviors, all contribute to reducing the likelihood of illness.

On one hand, enhancing the physical environment of primary care facilities can leverage their proximity to communities and local natural resources. Planting locally sourced greenery with mood-soothing properties in waiting areas, installing windows offering views of community green spaces or rivers, and playing gentle bird songs or stream white noise can alleviate medical anxiety while providing an inspirational setting for NQ cultivation. This creates a distinct contrast to the crowded, clinical settings of larger hospitals, serving as a key factor in attracting patients. Concurrently, optimizing service content involves integrating knowledge linking natural environments to health into routine care. For instance, hypertension patients could receive medication alongside walking programs utilizing community green spaces. This extends primary care beyond disease treatment to fostering healthy lifestyles – an area large hospitals struggle to cover due to heavy caseloads and limited service time. Such specialized offerings constitute a unique competitive advantage for primary care institutions, further alleviating economic and operational pressures on the healthcare system.

The most significant contribution of this study lies in two aspects. First, by introducing interaction from social support theory as an independent variable, it integrates social support theory with the elaborated possibility model. Using the central and peripheral pathways of the elaborated possibility model as mediating variables, it collectively constitutes the antecedents of attitudes. Results indicate that the integrated model provides a more comprehensive prediction of attitudes toward seeking medical care. Second, the study reveals the moderating role of perceived severity within the elaborated possibility model. It demonstrates that patients’ differing levels of self-perception intensity and emotional reactivity significantly influence their attitudes toward seeking medical care. This establishes an important boundary condition for the model in healthcare settings and lays a theoretical foundation for future efforts to cultivate patients’ health awareness.

In summary, this study offers a novel perspective on interpersonal persuasion and healthcare choice within medical service contexts, though certain limitations remain. Firstly, owing to data acquisition constraints, the sample comprised solely patients. Future research could incorporate clinician samples to facilitate multidimensional comparative studies, providing additional perspectives for a deeper, more comprehensive understanding of complex interpersonal persuasion processes. Secondly, patients’ health concept perceptions may also influence the persuasion process, particularly their natural quotient (NQ). Future research could further incorporate ‘NQ-oriented health perception’ as a core variable, exploring how healthcare institutions can utilize surrounding natural resources such as green spaces and rivers to conduct NQ education. This would cultivate patients’ health perceptions, reduce healthcare-seeking probability, and provide theoretical and practical guidance for alleviating the economic and psychological burdens associated with personal health treatment, while promoting the coordinated development of personal health management and the healthcare sector. Furthermore, constrained by socio-cultural differences, patients harbor stereotypical perceptions regarding the medical resources, technical capabilities, and competencies of tertiary hospitals versus community hospitals. Future research could compare the persuasion pathways between these settings to uncover patterns for developing more compelling communication strategies. This would encourage patients to rationally and proactively choose primary care institutions like community hospitals, thereby providing robust evidence for advancing the coordinated development of healthcare, ecology, and public health.

## Data Availability

The original contributions presented in the study are included in the article/Supplementary material, further inquiries can be directed to the corresponding author.
