# Supplementary material for: The impact of source credibility and information quality on healthcare consumers’ attitudes: the moderating role of perceived severity
Source: Front Public Health. 2025 Dec 4;13:1689496. doi: 10.3389/fpubh.2025.1689496 (PMC12711836; doi:10.3389/fpubh.2025.1689496)
Supplement: Supplementary file 1 [file Supplementary_file_1.docx]

## Robustness Test

In this study, methods such as the replacement test and exclusion of special samples were adopted to test the robustness of the model.

**（1）Bootstrap Test**

In this study, the Bootstrap method was used for robustness testing, using the Medcurve macro developed by Hayes and Preacher, with a set number of repeated samples of 5000 and a confidence interval of 95%. The statistical results are shown in Table 1 shows that the direct effect of interaction on attitude is significant, and the 95% confidence intervals of information quality and source credibility in the indirect effect of ‘interaction→attitude’ do not contain 0 (LLCI=0.106, ULCI=0.268; LLCI=0.194, ULCI=0.345), which indicates that information quality and source credibility play a significant role in mediating the effect of interaction on attitude. trustworthiness have a significant mediating role in interacting to influence attitudes, and the regression results of the robustness test are consistent with the path analysis regression results. Therefore, the conclusions of this study are robust.

**Table 1 Bootstrap test of information quality and source credibility in interaction affecting attitudes**

|  | Efficiency Value | Boot  standard error | 95% Confidence Interval | | Effect Size |
| --- | --- | --- | --- | --- | --- |
|  |  |  | Boot CI  lower limit | Boot CI  up limit |  |
| Total effect | 0.784 | 0.027 | 0.000 | 0.730 |  |
| Direct effect | 0.335 | 0.034 | 0.000 | 0.269 | 42.73% |
| Information Quality indirect effect | 0.180 | 0.041 | 0.106 | 0.268 | 22.96% |
| Source credibility indirect effect | 0.269 | 0.039 | 0.194 | 0.345 | 34.31% |

**(2) Special sample exclusion**

Excluding the ‘highly educated’ group. Considering the special characteristics of the highly educated group in terms of information perception, this study excludes the sample of ‘postgraduates and above’ for re-estimation. The results show that the path coefficients are all significant, as shown in Figure 1, and the model fitting results: χ2/DF=2.530, NFI=0.947, TLI=0.959, CFI=0.967, GFI=0.924, RESMA=0.065, which is a good fit. The results indicate that the mediating mechanisms of information quality and source credibility in the process of interaction's influence on attitudes are highly robust and the findings are reliable.

0.682***

0.798***

0.450***

0.434***

0.099^n^

**Figure 1 Robustness tests for excluding the ‘highly educated’ group**

Excluding groups with poorer health care resources. Considering the relatively limited medical resources in rural areas, the samples of ‘townships and neighbourhoods’ and ‘rural areas’ were excluded and re-estimated. The results show that the path coefficients are all significant, as shown in Figure 2 and the model fitting results: χ2/DF=2.483, NFI=0.950, TLI=0.963, CFI=0.969, GFI=0.929, RESMA=0.062, which is a good fit. The results of the above tests all indicate that the mediating mechanism of information quality and source credibility in the process of interaction's influence on attitudes is highly robust, and the findings are reliable.

0.699***

0.840***

0.571***

0.366***

0.087^n^

**Figure 2 Robustness tests excluding ‘town and neighbourhood’ and ‘rural’ groups**
